# Dynamic Mechanical Properties of PVC Plastics in the Formation of Microstructures with Novel Magnetostrictor

**DOI:** 10.3390/mi14040820

**Published:** 2023-04-06

**Authors:** Justas Ciganas, Algimantas Bubulis, Vytautas Jurenas, Paulius Griskevicius, Arvydas Palevicius, Sigita Urbaite, Giedrius Janusas

**Affiliations:** 1 Department of Mechanical Engineering, Kaunas University of Technology, Studentu 56, LT-51424 Kaunas, Lithuania; 2 Institute of Mechatronics, Kaunas University of Technology, Studentu 56, LT-51424 Kaunas, Lithuania

**Keywords:** magnetostrictor, hot-embossing, microstructure, plastics, dynamics

## Abstract

Molding in thermoplastic polymers using ultrasonic hot embossing technology is promising due to its high precision reproducibility. To understand, analyze and apply the formation of polymer microstructures by the ultrasonic hot embossing method, it is necessary to understand dynamic loading conditions. The Standard Linear Solid model (SLS) is a method that allows analyzing the viscoelastic properties of materials by representing them as a combination of springs and dashpots. However, this model is general, and it is challenging to represent a viscoelastic material with multiple relaxations. Therefore, this article aims to use the data obtained from dynamic mechanical analysis for extrapolation in a wide range of cyclic deformations and to use the obtained data in microstructure formation simulations. The formation was replicated using a novel magnetostrictor design that sets a specific temperature and vibration frequency. The changes were analyzed on a diffractometer. After the diffraction efficiency measurement, it was found that the highest quality structures were formed at a temperature of 68 °C, a frequency of 10 kHz, a frequency amplitude of 1.5 µm and a force of 1 kN force. Moreover, the structures could be molded on any thickness of plastic.

## 1. Introduction

Micromanufacturing technologies for micro-electro-mechanical systems (MEMS) are the key factor that determines the further development of the technology. Micro- or nanostructures could be produced by various technologies depending on the origin of the materials. For example, structures could be formed on electrically conductive materials using electroplating or electrodeposition technology [1]. Besides, biocompatible materials have their production technologies, such as two-photon polymerization nanolithography [2] or etching and direct-write techniques [3]. In addition, thermoplastic polymers are distinguished for their low melting temperature so that hot embossing technology could be used [4]. All these technologies have their advantages and disadvantages. All technologies may be limited by processed material, surface finishing, cost, and time consumption [5].

The production of microstructures from thermoplastic polymers is very promising because there is a very large selection of thermoplastic materials. These materials can be resistant to aggressive chemicals or temperatures up to 250 °C [6]. Moreover, additives incorporated into polymers could lead to electrical and thermal conductivity [7,8]. The mechanical properties of polymers depend on additives and chemical composition and can vary from elastic and stiff to brittle or hard [9]. Polymers can be optically transparent, colored, or matte. The optical properties can also be influenced by the geometry formed in the transparent bodies, usually formed using hot embossing technology [10]. Although polymers can be processed using other technologies such as milling [11] and laser processing [12]. The advantages of hot embossing technology are high precise repeatability and cheap mass production of structures [13].

The polymer is heated to the glass transition temperature using hot embossing technology. Then a mold is pressed into the polymer and held under pressure for a certain time. Later, the plastic is cooled until the required demolding temperature is reached, and the mold is separated from the plastic [5]. However, the disadvantages of this technology are that residual stresses are obtained, and the mold is not filled due to gas accumulations [14]. Due to gas accumulations, it is possible to remove gas using a vacuum chamber, but this is an additional process requiring time and special equipment [15]. High-frequency vibrations could be used to avoid bubbles and complex technological equipment [16].

Ultrasonic vibration energy could be used in various production technologies, such as welding plastics or metals and composite grinding materials or metals [15]. During the ultrasonic micro hot embossing technology, no heating is used, but due to vibrations and friction between the plastic and the mold, the plastic reaches the glass transition temperature and begins to flow into the mold [17]. The advantages of the technology are the possibility of forming structures with low load and obtaining large areas of structures with low internal stresses [18]. However, the main drawback is that the structures can be formed with a polymer thickness of 50 μm to 500 μm. With a thicker polymer, most of the energy is absorbed inside the polymer. Also, the process is influenced by the roughness of the mold—with a higher roughness, a greater amount of heat is released [15].

Usually, separate devices and complex structures are required to combine these two technologies [19]. Thus, in this article, microstructures were formed using a single device consisting of two processes: magnetostriction, which was responsible for the oscillation, and Fouquet currents, which generated the heating. The technological part of the device is not presented because the device is applied for patents: Lithuanian patent K158-89 and European patent K158-89 EP. To prove the performance of the designed novel magnetostrictor, experimental investigations were made to create microstructures at different temperatures and dynamic loads.

## 2. Materials and Methods

This section presents the viscoplastic properties of polymers at different temperatures and dynamic loads. Viscoplastic properties can be described by different methods. This study presents obtained storage and loss modulus from experimental data. Modulus values are converted to prony series coefficients using the “MCalibration” program.

**Materials.** This study chose thermoplastics polymer—polyvinyl chloride (PVC). The mechanical properties of PVC plastic were as follows, Young’s modulus was 3275 MPa, and the poison’s ratio was 0.4. In addition, multilinear isotropic hardening was selected and used from studies reported previously [20].

**Methods.** Dynamic mechanical analysis (DMA) was performed to determine the dynamic properties of viscoelastic solid material. In this research, a tensile test with excitation frequency was used. The experiments were done using the ISO 6721-4 standard. This standard describes the non-resonance method for polymers’ load with frequencies force from 0.01 Hz to 100 Hz. According to the standard, the output data of the experiment were storage modulus and loss factors.

**Figure 1:**
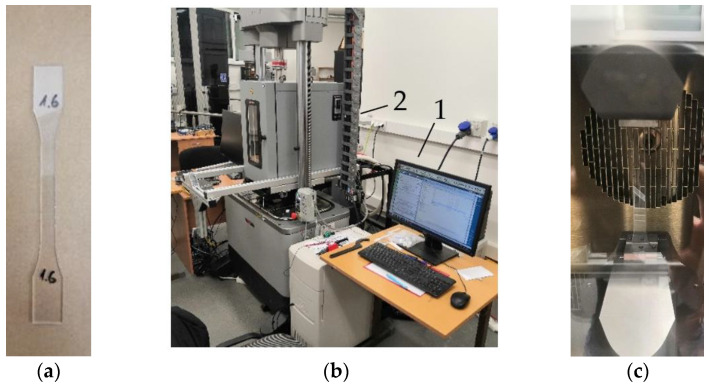
Tensile experiment: (**a**) Test specimen; (**b**) Testing machine Instron E10000: 1—control computer, 2—heating chamber and testing machine; (**c**) The test sample in the heating chamber.

A tensile machine (ElectroPuls E10000T Linear-Torsion, Instron, USA) was used for the dynamic tensile test. The experiment consisted of active elements of the stretching machine: thermal chamber, force transducer, clamp, test specimen, displacement, and vibrator transducer (Figure 1). The test specimen was prepared according to ISO 527-2 standards. In addition, each temperature and frequency range were maintained at the set temperature during the analysis according to ISO 6721-1.

A master matrix was used to create the microstructure in plastic, and it was fabricated using the methods of lithography and electroplating. The resulting microstructure was 2 µm wide, 4 µm periodic, and 1 µm deep. Scanning Electron Microscope (SEM) (S-3400N, Hitachi, Japan) and Atomic Force Microscope (AFM) (NT-206, Microtestmachines, Belarus) measurement techniques were performed on the obtained microstructure.

## 3. Results

### 3.1. Dynamic Mechanical Analysis

During the experimental dynamic mechanical analysis, a constant tension force was determined which was equal to 70 N, and the maximum dynamic amplitude was 80 N. Experiments were performed at a frequency of 0.1, 0.2, 0.5, 1, 2, 5, 10, 20, 30, and 40 Hz. The dynamic stretching parameters were obtained at 30–68 °C. After increasing the temperature to 70 °C, the specimen broke before the second dynamic cycle. The received data line contained the main data needed to calculate the dynamic characteristics: cycle elapsed time, stress, and displacement graph. The data were analyzed, and two graphs were obtained: amplitude of strain and stress. The discrepancy between these two graphs could be called “phase“ which increases with higher temperature. Finally, all the obtained data were recalculated and transformed into two constants (storage modulus and loss factors) at a specific temperature and excitation frequency:

(1) $$G^{\prime }=\frac{\sigma_{0}}{\varepsilon_{0}}cos\;\delta$$

(2) $$G^{\prime \prime }=\frac{\sigma_{0}}{\varepsilon_{0}}sin\;\delta$$

In the formula, *G*′ is storage modulus, *G*″ is a loss factor, *ε* is strain, *σ* is stress, and *δ* is a phase lag.

**Figure 2:**
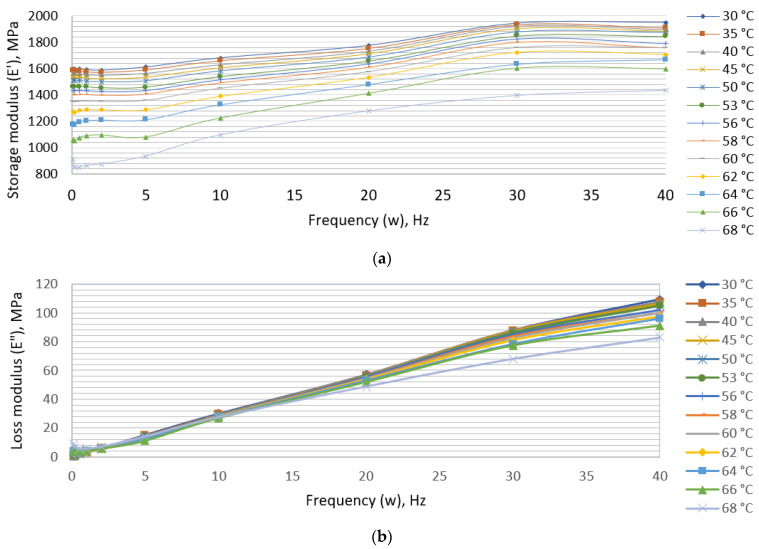
Experimental values: (**a**) Storage modulus; (**b**) Loss modulus.

The obtained parameters of storage modulus and loss factors at different temperatures are presented in Figure 2. The obtained storage modulus and loss factors curves reflect the material’s behaviour at up to 40 Hz.

Due to the limitations of the Instron stretching machine, only low-frequency range data could be analyzed, but the experiment required extended analysis in a higher frequency range. The principle of time—temperature superposition (TTS), or frequency—temperature superposition, was used to expand this type of analysis. This analysis provides an opportunity to determine the temperature dependence of the rheological behavior of thermoplastic polymer and to expand the limits of unknown temperature frequencies [21].

Using the principle of superposition, a curve was obtained. To analyze the curve at high frequency, the curve must be extrapolated using the fit function with coefficients. Often, in this type of analysis, a master curve is fitted to a sigmoidal function to get storage modulus. To get storage (*E*′) and loss (*E*″) modulus, it needs to approximation with Kramers-Kronig relations after using sigmoidal fit coefficients [22].

(3) $$E^{\prime }\left(w\right)=a\cdot \tanh\left(b\left(\log\left(w\right)+c\right)\right)+d$$

(4) $$E^{\prime \prime }\left(w\right)=\frac{\pi \cdot a\cdot b}{2}\mathrm{sech}(b{\left(log\left(w\right)+c\right)}^{2}$$

In the formula, *a*, *b*, *c* and *d* are fit coefficients, *log*(*w*) is the natural logarithm. After extrapolation, fit function coefficients with the goodness of fit were obtained. The results are presented in Table 1.

**Figure 3:**
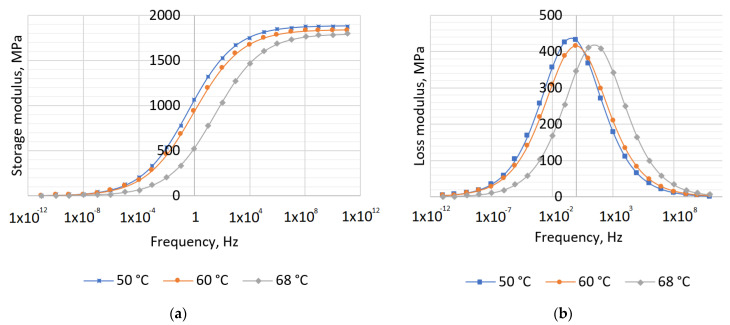
Master curves of (**a**) Storage modulus; (**b**) Loss modulus.

After receiving the coefficients, the values were inserted into the equation, and the master curve of loss and storage modulus was defined in a wide range of vibrations. The resulting curves are presented in Figure 3.

Simulations of vibration and viscoelastic hot embossing process. Finite element modeling was used for further data processing and microstructure variation using ultrasonic hot embossing technology. The main parameters of the mechanical analysis are presented in Table 1. However, to evaluate the vibrations, it was necessary to recalculate the storage and loss modules through Prony coefficients. Storage and loss modules with constants and equations can usually be calculated from the Prony coefficients [23].

(5) $$E^{\prime }\left(w\right)=E_{0}\left[1-\sum_{i=1}^{N}g_{i}\right]+E_{0}\sum_{i=1}^{N}\frac{g_{i}T_{i}^{2}w^{2}}{1+T_{i}^{2}w^{2}}\;\;\;\;\;$$

(6) $$E^{\prime \prime }\left(w\right)=E_{0}\sum_{i=1}^{N}\frac{g_{i}T_{i}^{2}w^{2}}{1+T_{i}^{2}w^{2}}$$

Prony series coefficients were calculated after using the available loss and storage modules. *N* values and guess values for *E*_0_, *g_i_*, and *T_i_* were chosen to find the values. After calculating the *G*′ and *G*″ values, they were compared with the experimental ones. Moreover, the R^2^ values were calculated, and the cycle was repeated until the R^2^ value reached the closest value of 1. Then, 23 Prony series terms were chosen to obtain the closest value to the experimental curve. The resulting constants were used in the finite element model.

**Figure 4:**
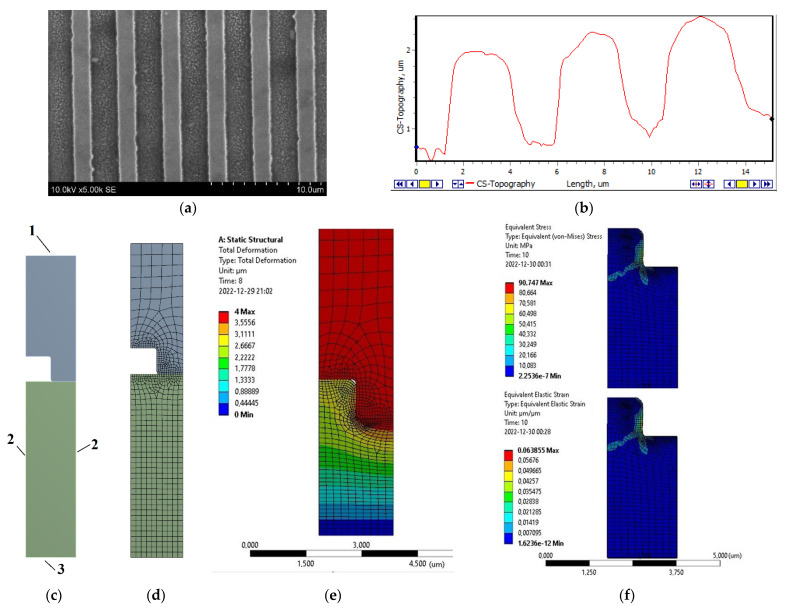
Images of the master matrix: (**a**) SEM image of the master matrix, (**b**) AFM topography surface profiles; Finite element analysis: (**c**) model with boundary conditions: 1—remote displacement, 2—frictionless support, 3—fixed support; (**d**) model with mesh; (**e**) total deformation; (**f**) equivalent stress and equivalent elastic strain.

Finally, the finite element model was created and analyzed in Ansys software. To optimize the simulation, the model was selected as 2D type and only half of one groove model was selected, indicating its thickness—1 µm. The geometry consists of two separate bodies—the mold and the plastic, whose geometry and constraints are shown in Figure 4. The mold material was a nickel, the same as the master grating plate, which was further used in the embossing. A friction coefficient of 0.2 was chosen at the point of body contact. The model was decomposed into 1704 finite elements. A nonlinear adaptive region was applied to the model for a more accurate result, which recalculates the grid in case of large deformations. After using the remote displacement support, the mold was given a movement of up to 4 µm. Then the mold was supported (1 s) and retracted in 1 s. The images obtained using SEM and AFM measurements of the master matrix and deformed body are presented in Figure 4.

To check whether the grid was suitable, a grid-independent verification study was chosen. Strain and stress results stabilized at the medium grid. Increasing the grid further did not have a significant effect. Therefore, it was possible to provide that the mesh could be used in further calculations. The grid-independence check is presented in Table 2.

**Figure 5:**
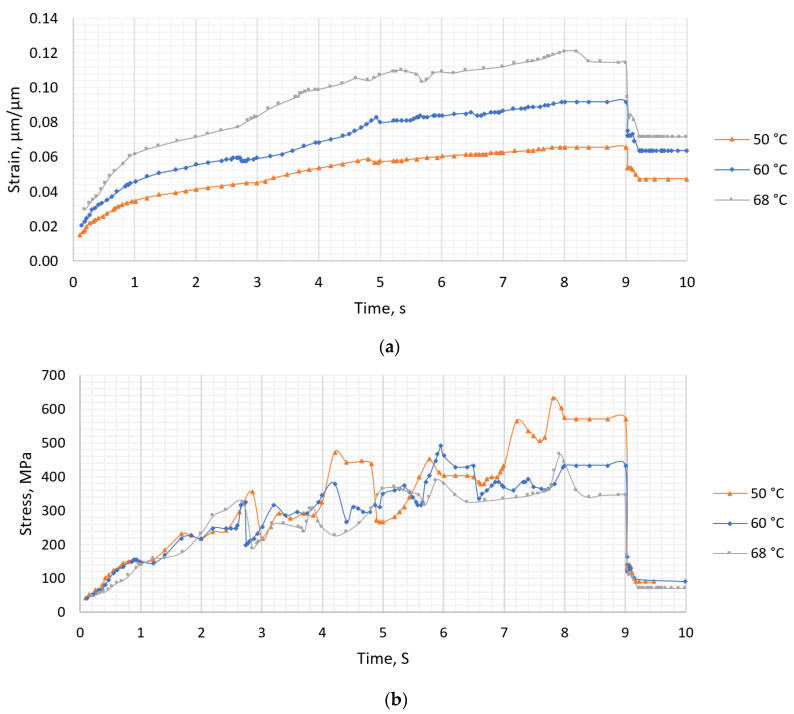
Results of simulation: (**a**) Equivalent elastic strain; (**b**) Equivalent stress.

After the simulations, 2 diagrams were formed showing the changes in strain and stress. The value of the strain curve increased at higher temperatures and the same excitation frequency. At 8 s and a temperature of 68 °C, the plastic was deformed, and from the stress graph, it can be seen that the stress was decreasing. The residual stress decreased at higher temperatures. The graphs of the results are presented in Figure 5.

### 3.2. Thermal Imprint with Magnetostrictor

The hot embossing process has three main stages: heating, embossing, and cooling. Often, the plastic is heated to the embossing temperature, and then the embossing process is carried out for a set time. After removing the load, a cooling process is carried out. In our case, two different stages were removed, and one was obtained using a magnetostrictor. Finally, the mold was heated, and vibrations were used then. This results in local heating with vibrations.

**Figure 6:**
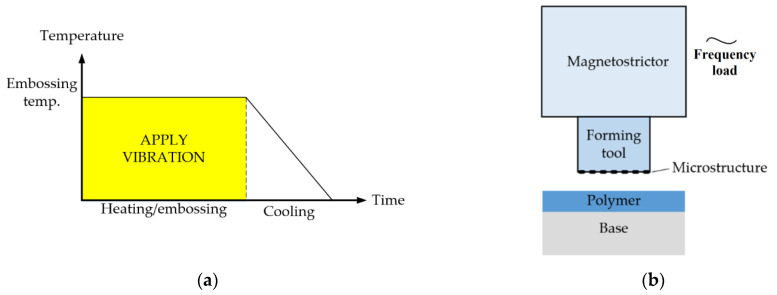
(**a**) Temperature curves for hot embossing procedures using a magnetostrictor; (**b**) Principle of operation of the magnetostrictor.

**Figure 7:**
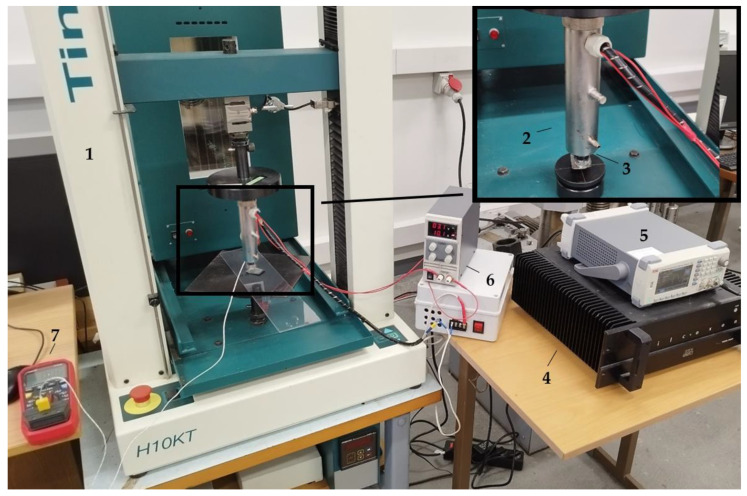
Microstructure forming equipment: 1—Tensile test machine H10KT (Tinius Olsen, Horsham, JAV), 2—magnetostrictor, 3—forming grid, 4—amplifier PA8HF (Wilcoxon, Gamlingay, UK), 5—signal generator UTG2025A (UNI-T, Hong Kong, China), 6—power source KPS3010D (wanptek, Shen Zhen, China), 7—thermometer UT161D (UNI-T, Hong Kong, China).

As shown in Figure 6, the forming equipment comprised a magnetostrictor, a forming tool with a microstructure, polymer plates and a base. As shown in Figure 7, the vibration system also consisted of a signal generator and an amplifier, which could increase the signal power. Vibrations whose amplitude could reach 4 µm were obtained. Then, the forming tool started to heat up according to the set duration.

**Figure 8:**
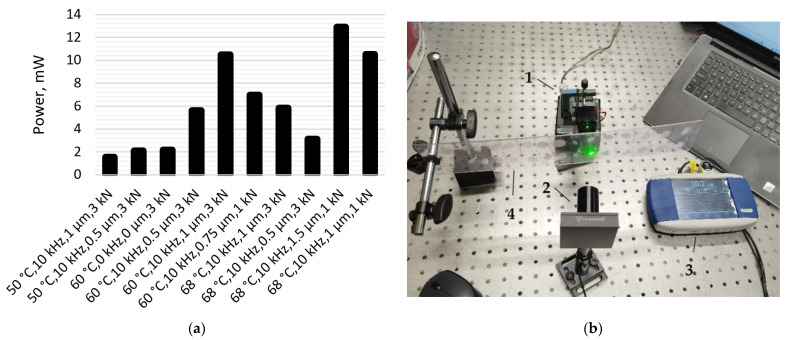
(**a**) Diffraction efficiency measurement results; (**b**) measurement equipment: 1—laser (OX-MZ5201, OXLasers, China), 2—laser detector (11UP12, Gentec, Canada), 3—energy monitor (201235, Gentec, Canada), 4—grid.

The structures were formed by changing the temperature, forming force, vibration amplitude and frequency. Indentation with the same parameters was repeated 3 times to obtain more accurate results in further measurements. The quality of the structure could be evaluated in various ways, but diffraction is used in this study. A green (525 nm) laser and a high-power laser detector with an energy monitor were both used in the experiment. The experimental equipment is presented in Figure 8b. Each peak was recorded on a computer and processed.

During the diffraction measurements, 30 samples were measured (3 using the same parameters). Diffraction efficiency was calculated using averages of 3 identical samples. The best relative diffraction efficiency (RDE) was at 68 °C, 10 kHz excitation frequency with 1.5 µm deformation amplitude and 1 kN load. The best RDE was at 60 °C with an excitation frequency of 10 kHz with 1 µm deformation amplitude and a load of 3 kN (Figure 8a).

## 4. Discussion

The formation of microstructures with hot embossing technology is a fast way to form a microstructure on a large area. To ensure high-aspect-ratio microchannels in a large area, it is necessary to use vibration as an assisted in forming process. Having a more complex geometry and searching for optimal parameters in the forming process, having a final element model with material properties where vibration could be applied as an additional tool helps produce microstructures. The results confirmed that the use of vibrations improves the quality of the grid. Therefore, it is possible to form microstructures at lower temperatures and obtain the same or even better optical properties.

In this case, the optimal parameters for forming the main matrix are 68 °C, 10 kHz excitation frequency with 1.5 µm deformation amplitude and 1 kN load. After changing the parameters of the main matrix, the parameters of the derivative can change for several reasons. In particular, it may take longer for the plastic to flow into the mold due to the change in volume and heat transfer contact area. Second, when the lattice parameters change, the theoretical relative diffraction efficiency values also change, which may affect the required forming parameters. Third, a higher forming temperature or forming force could be selected for more complex geometries to shorten the forming time, but this requires further investigation. Finally, these molding parameters may only apply to PVC plastic, and the molding parameters for other plastics may vary.

## 5. Conclusions

Using DMA material analysis, viscoelastic materials such as thermoplastics polymers can be analyzed with a finite element model. DMA measurements showed that the storage modulus increased with increasing forming frequency regardless of the forming temperature. The loss module changed unevenly. It increased at a low frequency. As the frequency increased, the loss module decreased. These DMA data were extrapolated using viscoelastic proxy coefficients. Using a finite element model, it was possible to find optimal forming parameters using a new magnetostrictor technology. Using the results of the finite element model and selecting the forming parameters, 10 different lattices were embossed. After the diffraction efficiency measurement, the results showed that it is best to form the structure with the new magnetostrictor technology at a temperature of 68 °C, a frequency of 10 kHz and a frequency amplitude of 1.5 µm and a force of 1 kN force. A notable advantage of this technology is that the structure can be formed on any thickness of plastic.

## 6. Patents

Applied for patents: Lithuanian patent K158-89 and European patent K158-89 EP.

## Figures and Tables

**Table 1:** Fit function coefficients with the goodness of fit.

Temperature, °C	a	b	c	d	R^2^
50	942	0.2966	0.437	942	0.997
60	920.6	0.2879	0.05601	920.6	0.998
68	898.5	0.2971	−1.473	898.5	0.996

**Table 2:** Study results of grid-independent verification.

	Amount of Elements	Strain, µm/µm	Stress, MPa
Coarse	791	0.12095	701.15
Medium	1704	0.12412	421.78
Fine	6066	0.12129	431.74

## Data Availability

Not applicable.
